# Tooth loss and associated factors during periodontal maintenance care in young adults with Grade C Periodontitis at a tertiary care center in Kathmandu, Nepal

**DOI:** 10.1371/journal.pone.0352255

**Published:** 2026-06-26

**Authors:** Khushboo Goel, Sirjana Dahal

**Affiliations:** 1 Department of Periodontics, Tribhuvan University, Institute of Medicine, Maharajgunj Medical Campus, Dental Teaching Hospital, Kathmandu, Nepal; 2 Department of Community Dentistry, Tribhuvan University, Institute of Medicine, Maharajgunj Medical Campus, Dental Teaching Hospital, Kathmandu, Nepal; Debreceni Egyetem, HUNGARY

## Abstract

**Background:**

This study aimed to evaluate tooth loss attributable to periodontal disease during periodontal maintenance care (PMC) in young adults with Grade C Periodontitis and to identify factors associated with tooth loss.

**Methods:**

A total of 63 patients (mean age: 29.49 ± 4.65 years) were included. All patients received non‑surgical periodontal therapy using a comprehensive full‑mouth disinfection protocol with adjunctive systemic antibiotics as part of active periodontal therapy (APT), delivered through an individualized treatment approach. Clinical assessments were performed after completion of APT (T1) and during PMC, T2. Multivariable Firth-penalized Cox proportional hazards regression was applied to identify factors associated with time to tooth loss.

**Results:**

A total of 1,709 teeth (1,228 non-molars, 481 molars) were analyzed. Over a mean follow‑up period of 60 ± 2 months, 15 teeth were lost, corresponding to an overall tooth loss rate of 0.88% (0.048 teeth/patient/year). Bone loss <60%, Molar‑Incisor Grade C Periodontitis, and adherence to PMC were associated with greater tooth retention. The overall regression model was statistically significant (likelihood ratio test p = 0.0016). Bone loss exceeding 60% was associated with a significantly increased hazard of tooth loss (HR: 14.83; 95% CI: 1.98–1896.79; *p* = 0.003), whereas compliant patients with PMC showed a significant protective association (HR: 0.29; 95% CI: 0.10–0.79; *p* = 0.016).

**Conclusion:**

Tooth loss during PMC was minimal over 5 years. Severe periodontal bone loss and non‑compliance with maintenance care were associated with increased tooth‑loss risk. Long‑term retention of most teeth appears achievable through conservative periodontal management in young adults with Grade C Periodontitis.

## Introduction

Periodontitis, recognized as the sixth most prevalent disease globally, arises from dysregulation of the host immune-inflammatory response and constitutes one of the primary etiological factors for tooth loss, leading to diminished esthetics, impaired function, and a compromised quality of life [1,2]. The management of periodontitis necessitates the implementation of active periodontal therapy (APT), which encompasses both supra- and subgingival scaling, root planing, and various surgical interventions supported by adjunctive measures that are efficacious in halting disease progression and stabilizing alveolar bone levels. Following the stabilization of tissue, periodontal maintenance care (PMC) is instituted, which aims to prevent any further progression of the disease through supra- and subgingival mechanical debridement, the management of established risk factors, and the modification of patient behavior [3–5]. Grade C Periodontitis particularly represents a severe manifestation of multifactorial inflammatory periodontal disease. Children and young adults in the permanent dentition phase, particularly those aged below 35 years, exhibit vulnerability to the rapid deterioration of periodontal attachment and bone, leading to early tooth loss in their current condition or in the near future if appropriate diagnostic and therapeutic measures are not implemented [6–8]. The treatment protocol for Grade C Periodontitis includes both mechanical and surgical periodontal therapies, supplemented by the administration of adjunctive antibiotics. A recent meta-analysis indicates that the long-term success rate for the preservation of teeth affected by Grade C Periodontitis is comparable to that observed in cases of generalized chronic periodontitis [9].

The success of any periodontal therapy is assessed through arrest in the rate of disease progression and the long-term retention of teeth. Existing literature suggests that retention of teeth or prevention of tooth loss in severe periodontitis constitutes a feasible objective with essential prerequisites such as regular consultations with specialists and the implementation of a meticulously tailored plan that considers the patient’s periodontal baseline severity, susceptibility, medical history, and response to the APT protocol [10,11]. Furthermore, the maintenance of periodontal health via PMC may yield cost-effectiveness when compared to prosthetic alternatives for the replacement of lost teeth [12]. Given the low prevalence of stage III/IV, Grade C Periodontitis cases, data pertaining to treatment outcomes and tooth survival over extended follow-up periods remain scarce [13–15], particularly with follow-up durations exceeding five years [9]. Hence, this study aims to evaluate tooth loss attributed to periodontal disease in patients undergoing conservative non-surgical periodontal therapy during PMC (T1 – T2) in young adults within a university context, and identify factors associated with tooth loss.

## Methods

**Patients:** This retrospective investigation included 63 patients diagnosed with Grade C Periodontitis, who were treated from 21/01/2019–29/09/2025 within the Department of Periodontics at Dental Teaching Hospital, Institute of Medicine, Tribhuvan University. On average, 3–4 patients each month, classified with stage III/IV, Grade C Periodontitis presented to the department. A complete enumeration technique was employed for the recruitment of study participants. Ethical approval for this study was obtained from Institutional Review Committee (IRC) of Institute of Medicine (IOM), Tribhuvan University (TU) with Ref No. 100/082/083. All experiments were conducted in accordance with the Declaration of Helsinki and relevant institutional guidelines and regulations. Written informed consent was obtained from all participants prior to their participation as routine departmental procedure. The data were accessed for research purposes from 6/10/2025–13/10/2025 from department archives.

Inclusion criteria included were initial aged between 14–35 years, no known systemic disease (apart from diabetes mellitus) that might influence the progression of periodontal disease, periodontally compromised patients exhibiting stage III/IV, Grade C Periodontitis [16]; active or current smokers who have been smoking more than five cigarettes per day for the preceding two years or longer; patients who underwent treatment involving a non-surgical modified full-mouth disinfection protocol [17] combined with antibiotic prescriptions subsequent to January 2019 [18]; patients who experienced tooth loss exclusively attributable to periodontal disease; and those who consented to participation and signed informed consent documents. The exclusion criteria encompassed patients diagnosed with gingivitis, stage I/II, Grade A/B periodontitis, individuals requiring additional surgical interventions for periodontal maintenance, and those with third molars.

### Clinical examination

Data were collected using pre‑tested semi‑structured questionnaires administered through face‑to‑face interviews in the local language. Information obtained included demographic characteristics, medical history, and adverse oral habits. This was followed by a comprehensive periodontal clinical examination. Periodontal examinations were performed by calibrated dental hygienists under the supervision of an experienced periodontist (Dr. K. G.). All examiners underwent calibration training in periodontal disease assessment. Inter‑examiner reliability for periodontal parameters was assessed using weighted kappa statistics, with values exceeding 0.85, indicating strong agreement and measurement reliability.

Periodontal diagnosis was established according to the 2018 Classification of Periodontal and Peri‑Implant Diseases and Conditions. Given the strong association of the new classification with tooth loss due to periodontitis during both active periodontal therapy (APT) and supportive periodontal therapy (SPT) [19], patients were diagnosed with Stage III or IV Grade C Periodontitis at baseline. Stage III/IV periodontitis was defined by interdental clinical attachment loss (CAL) ≥5 mm at the site of greatest loss, radiographic bone loss extending to the middle or apical third of the root, and the presence of complexity factors, including probing depths ≥6 mm, vertical bone loss ≥3 mm, Class II or III furcation involvement, moderate to severe ridge defects, and tooth mobility of grade ≥2 [20]. Following APT, the baseline prognosis of affected teeth ranged from fair to poor or hopeless [21]. Grade C Periodontitis was defined by indirect evidence of rapid disease progression, indicated by a bone loss‑to‑age ratio >1.0. Bone loss was further categorized as ≥50% to <60% and ≥60%. For subgroup analysis, Grade C Periodontitis was categorized into molar‑incisor presentation Grade C Periodontitis (MI‑GC‑P), characterized by involvement of at least the first molars and/or incisors and affecting fewer than 30% of remaining teeth (maximum of seven teeth), and generalized Grade C Periodontitis (G‑GC‑P), defined by involvement of more than 30% of remaining teeth (at least eight teeth), including at least three permanent teeth other than molars and incisors [22,23].

A complete periodontal examination entailed the recording of PDs, CAL and FI for first premolars and multi-rooted teeth, as well as the assessment of tooth mobility. The UNC-15 Color-Coded Probe (Hu-Friedy, Chicago, IL, USA), featuring a black band for each millimeter up to 15 millimeters, was employed to measure six sites on each tooth, including the mid-buccal, distobuccal, mesio-buccal, mesio-lingual, distolingual, and mid-lingual areas. The pocket depth and clinical attachment level were recorded by measuring the distance from the free gingival margin and cemento-enamel junction to the base of the pocket, respectively. Furcation involvement for molars and premolars was evaluated in accordance with the methodology established by Glickman et al [24]. Tooth mobility was assessed and classified into degrees 0–3, consistent with the criteria set forth by Lindhe and Nyman (1977) [25]. For the present study, number of teeth with FI was documented along with count of teeth displaying degree II and III mobility at time points T1 and T2.

### Active Periodontal Therapy (APT) and Periodontal Maintenance Care (PMC)

All 63 patients remain under continuous observation and are scheduled for biannual evaluations to ascertain treatment requirements. Documentation of tooth loss and periodontal recordings was conducted at baseline, at the conclusion of APT (T1), and at the end of 60 ± 2 months of PMC (T2). The recordings from T1 and T2 were incorporated into the subsequent analysis. At baseline, all participants were provided with oral hygiene education and underwent supragingival scaling within a two-week timeframe. During the second phase of periodontal intervention, APT encompassed a non-surgical full-mouth disinfection protocol (FMDis) [17], executed four weeks post-baseline. This FMDis was augmented with systemic antibiotics (amoxicillin 500 mg and metronidazole 400 mg for a duration of eight days) [26]. Furcation involvement (FI) was addressed through the use of hand instruments and ultrasonic scaler tips [27].

**Fig 1:**
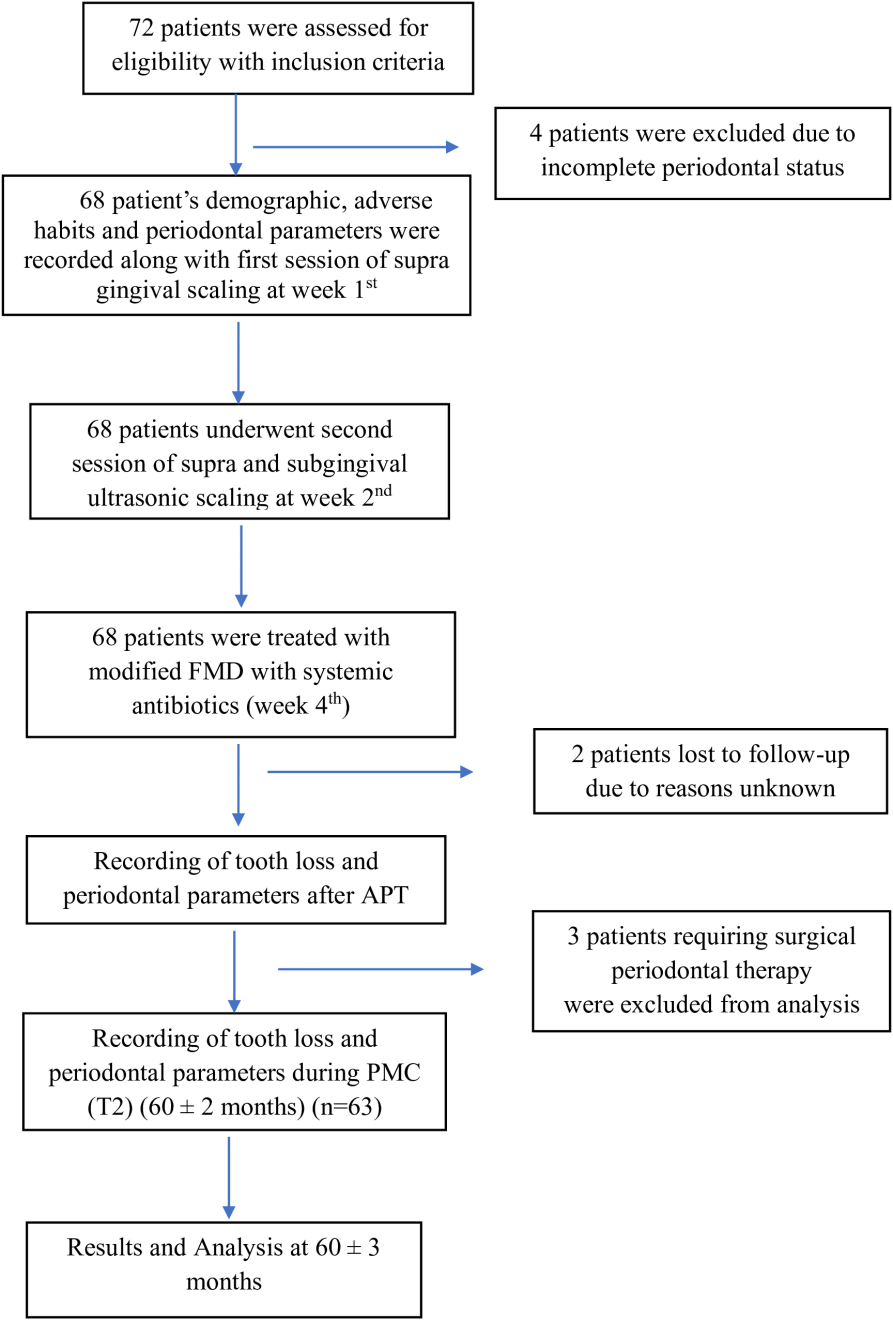
Patient flow diagram including the timings of assessments. Fig 1 shows, step-by-step progress (enrollment, intervention, follow-up) of patients including measurements at baseline and follow-ups during the study period.

Additional interventions, such as endodontic procedures and the splinting of mobile teeth, were conducted. Patients received reinforcement for maintaining adequate oral hygiene, specifically customized to include recommendations for tooth brushing and the supplementary use of interdental brushes. For areas challenging to access, alternative interdental cleaning devices were recommended. Smoking cessation counseling was provided with positive reinforcement for patients who smoke. Patients who reported significant gingival recession following APT were not subjected to pocket elimination or surgical procedures (including regenerative and other resective surgeries). In instances where PDs exceeded 7 mm, along with the presence of abundant keratinized tissue, bleeding on probing, and furcation involvement, access flap surgeries and tunneling procedures were performed but excluded from the final analysis. PMC entailed the re-instruction of oral hygiene practices, re-motivation, and professional re-instrumentation and irrigation of residual pockets by a qualified periodontist. All patients underwent examination every three months during the first year, with treatment approaches tailored according to the individual risk of recurrence or progression [28]. Patients were categorized based on their adherence to scheduled maintenance visits. Regular compliers were defined as patients who attended quarterly PMC visits during the first year and subsequently adhered to annual or biannual recall visits in the following years. Irregular compliers were those who attended maintenance care inconsistently, failing to adhere to quarterly visits during the first year and/or annual follow‑up visits in subsequent years [29,30].

### Statistical analysis

Data were entered into Microsoft Excel (2010) and analyzed using the Statistical Package for Social Sciences (SPSS), version 24 (IBM Corp., Armonk, NY, USA). Kolmogorov-Smirnov (K-S) test was used for checking the normality distribution of data. Continuous variables were summarized using mean and standard deviation for normally distributed data and median with interquartile range for non‑normally distributed data. Categorical variables were summarized using frequencies and percentages. Within‑subject comparisons between conclusion of APT, T1 and follow‑up measurements (PMC, T2) were conducted using paired *t*‑tests for normally distributed variables and Wilcoxon signed‑rank tests for skewed distributions. Associations between predictive factors and tooth loss were assessed using the chi‑square test or Fisher’s exact test, as appropriate. Independent *t*‑tests were used to compare continuous variables across groups defined by tooth retention status at the 5‑year follow‑up. Statistical significance was set at *p* < 0.05. Further, statistical analyses were performed using R version 4.3.1 (R Core Team, 2024). The following R packages were utilized: survival (version 3.5–5) for survival analysis and Kaplan-Meier estimation, survminer (version 0.4.9) for graphical presentation of survival curves, coxphf (version 1.13.2) for Firth penalized Cox regression, ggplot2 (version 3.4.4) for custom visualizations, and dplyr(version 1.1.2) for data manipulation.

## Results

A total of 63 patients (40 males, 23 females), diagnosed with MI-GC-P (n = 23) or G-GC-P (n = 40), exhibited an average age of 29.49 ± 4.65 years were included in this study. Seventeen of these patients were active smokers, and none had a diagnosis of diabetes. All patients underwent recurrence therapy, and 39 patients (61.9%) regularly participated in PMC. Further characteristics of the study population are delineated in Table 1.

**Table 1:** Characteristics of study population at conclusion of APT (T1, n = 63).

Variables	MI-GC-P (n = 23)	G-GC-P (n = 40)	Total (n = 63)
Mean Age in years (SD)	27.48 (4.87)	30.65 (4.14)	29.49 (4.65)
Gender n (%)			
Females	7 (30.4)	16 (40.0)	23(36.5)
Males	16 (69.6)	24 (60.0)	40 (63.5)
Smoking n (%)			
Active	4 (17.4)	13 (32.5)	17 (27.0)
Non-smokers	19 (82.6)	27 (67.5)	46 (73.0)
PMC n (%)			
Regular	12 (52.2)	27 (67.5)	39 (61.9)
Irregular	11 (47.8)	13 (32.5)	24 (38.1)
Periodontal bone loss n (%)			
≥50 to < 60%	13 (56.5)	3 (7.5)	16 (25.4)
≥60%	10 (43.5)	37 (92.5)	47 (74.6)

MI-GC-P: Molar-Incisor Grade C Periodontitis; G-GC-P: Generalized Grade C Periodontitis; PMC: Periodontal Maintenance Care.

Table 2 presents the distribution of tooth loss of study participants at the conclusion of APT and the commencement of PMC. In total, 1709 teeth were treated. Prior to the initiation of periodontal therapy, at baseline, patients had experienced the loss of 55 teeth (3.14%, third molars excluded) upon their presentation to the Department of Periodontics. Altogether, 27.13 teeth were present at the onset of PMC. Throughout APT, no extractions were performed of questionable and hopeless teeth. During the five-year duration of PMC, 15 teeth (0.88%, equivalent to 0.048 teeth per patient per year) were extracted for periodontal reasons. Each patient experienced the loss of only a single tooth during PMC. An increase number of teeth loss was observed in G‑GC‑P, cases (13,1.2%,0.065 tooth/patient/year) in comparison to MI‑GC‑P, cases (02, 0.31%, 0.017tooth/patient/year). Comparable teeth were lost in both maxilla and mandible jaws with no significant predominance observed. A greater number of molars (n = 9, 1.87%, 0.02 molars lost/patient/year) were lost relative to non-molar teeth (n = 6, 0.48%, 0.019 non-molars lost/patient/year) over the 5-year span. Notably, none of the premolars were lost among the 63 patients.

**Table 2:** Distribution of number of tooth loss of study population at beginning of PMC (T1) and in 5 years of PMC (T2) (n = 63).

Variables	MI-GC-P (n = 23)	G-GC-P (n = 40)	Total (n = 63)
No. of teeth T1 T2	636 634	1073 1060	1709 1694
Tooth loss	2	13	15
Maxilla T1 T2	315 315	537 530	852 845
Tooth loss	0	7	7
Mandible T1 T2	321 319	536 530	857 849
Tooth loss	2	6	08
Tooth type Non-Molar T1 T2 Tooth loss Molars T1 T2 Teeth loss	454 453 1 182 181 1	774 769 5 299 291 8	1228 1222 6 481 472 9

MI-GC-P: Molar-Incisor Grade C Periodontitis; G-GC-P: Generalized Grade C Periodontitis.

Table 3 presents the comparison of predictive factors regarding the number of teeth retained at T1 and T2. There was no significant association identified between gender and smoking with respect to the retention of teeth present at T1 and T2 (p > 0.05). The regularity of patient visits during APT (T1) did not significantly influence the number of teeth, however, patients who consistently attended their appointments during PMC (T2) retained a significantly greater number of teeth compared to those who paid irregular visits for maintenance care. Patients with bone loss exceeding 60% demonstrated a significantly reduced number of teeth, both initially at T1 and during the follow-up at PMC. Patients diagnosed with MI-GC-P exhibited greater tooth retention than those with G-GC-P.

**Table 3:** Comparison of predictive factors regarding number of teeth present at beginning of PMC (T1) and in 5 years of PMC (T2).

Variables	Time of evaluation	Category	Number of teeth Mean (SD)	t	p-value*
Gender	T1	Male	27.10 (1.24)	−0.283	0.779
		Female	27.17 (0.83)		
	T2	Male	26.85 (1.35)	−0.335	0.739
		Female	26.96 (0.93)		
Smoking	T1	Absent	27.24 (0.95)	1.545	0.128
		Present	26.75 (1.44)		
	T2	Absent	27.02 (1.11)	1.692	0.096
		Present	26.44 (1.41)		
PMC	T1	Irregular	26.88 (1.51)	0.110	0.155
		Regular	27.28 (0.72)		
	T2	Irregular	26.50 (1.56)	0.118	**0.044**
		Regular	27.13 (0.86)		
Periodontal bone loss	T1	≥50 to < 60%	27.75 (0.45)	2.760	**0.008**
		≥60%	26.91 (1.18)		
	T2	≥50 to < 60%	27.75 (0.45)	3.613	**0.001**
		≥60%	26.60 (1.25)		
Grade C Periodontitis	T1	MI-GC-P	27.65 (0.57)	3.062	**0.003**
		G-GC-P	26.83 (1.22)		
	T2	MI-GC-P	27.57 (0.66)	3.705	**<0.001**
		G-GC-P	26.50 (1.28)		

*Independent t test; MI-GC-P: Molar-Incisor Grade C Periodontitis; G-GC-P: Generalized Grade C Periodontitis; PMC: Periodontal Maintenance Care.

Table 4 shows the association of predictive factors with teeth loss. No statistically significant association of teeth loss was detected during the periodontal maintenance care (T1-T2) concerning the assessed factors, regardless of smoking status, frequency of dental visits, bone loss, or the existential diagnosis of Grade C Periodontitis.

**Table 4:** Association of predictive factors with tooth loss due to periodontal disease (n = 63).

Variables	Category	Teeth loss n (%)		p-value
Absent	Present
Gender	Male	30 (75.0)	10 (25.0)	>0.99*
	Female	18 (78.3)	5 (21.7)	
Smoking	Absent	35 (76.1)	11 (23.9)	>0.99^a^
	Present	13 (76.5)	4 (23.5)	
PMC	Irregular	17 (70.8)	7 (29.2)	0.434*
	Regular	31 (79.5)	8 (20.5)	
Periodontal bone loss	≥50 to < 60%	13 (81.3)	3 (18.8)	0.740 ^a^
	≥60%	35 (74.5)	12 (25.5)	
Grade C Periodontitis	MIPP	17 (73.9)	6 (26.1)	0.748*
	Generalized	31 (77.5)	9 (22.5)	

*Chi square test, ^a^Fisher’s exact test, MIPP: Molar-incisor pattern periodontitis; GC-P; PMC: Periodontal Maintenance Care.

Table 5 provides a comparative analysis of periodontal characteristics of study population at T1 and T2. A statistically significant decrease in the mean PDs and CAL were documented. Significant improvements in furcation involvement and mobility levels were observed (p < 0.05).

**Table 5:** Comparison of periodontal characteristics of study population at T1 and T2 (n = 63).

Characteristics	T1	T2	p-value
Mean PDs (SD)	4.41 (1.03)	2.49 (0.70)	<0.001*
Mean CAL (SD)	5.16 (1.40)	3.34 (1.10)	<0.001*
FI Median (IQR)	3 (2-5)	2 (1-3)	<0.001^a^
Mobility Median (IQR) Degree I/II Degree III	5 (3-7) 3 (2-4)	2 (1-4) 1 (1-3)	<0.001^a^ <0.001^a^

*Paired t test, ^a^Wilcoxon signed ranked test, PD: probing depths, CAL: clinical attachment loss, FI: furcation involvement.

Table 6 shows the penalized Cox regression model to identify factors associated with time for tooth loss. The number of factors were reduced to fit the model, and to represent the data which would be clinically relevant and statistically appropriate. During the follow-up period, 15 participants (23.8%) experienced tooth loss, whereas 48 (76.2%) were censored. The overall model was statistically significant (likelihood ratio test p = 0.0016), indicating good explanatory ability. Periodontal bone loss exceeding 60% was significantly associated with an increased hazard of tooth loss (HR: 14.83, 95% CI: 1.98–1896.79, p = 0.003). Conversely, PMC demonstrated a significant protective effect (HR: 0.29, 95% CI: 0.10–0.79, p = 0.016). The proportional hazards assumption was evaluated using Schoenfeld residuals. No significant violation was observed for individual covariates or globally (global test p = 0.60). However, given the limited number of events, these results should be interpreted with caution.

**Table 6:** Multivariable Firth penalized Cox proportional hazards analysis of factors associated with tooth loss.

Variables	HR	95% CI	p-value
Periodontal bone loss >60%	14.83	1.98–1896.79	**0.003**
PMC	0.29	0.10–0.79	**0.016**

PMC: Periodontal Maintenance Care; Hazard Ratios (HR) and p-values or 95% CI indicate higher or lower risk of tooth loss.

Table 7 depicts the five-year tooth survival and cumulative incidence using Kaplan–Meier analysis in Patients with Periodontal Disease. The five-year cumulative incidence of tooth loss was 23.8%. The Kaplan-Meier survival probability at 60 months was 0.76 (95% CI: 0.64–0.86). The annual tooth loss rate was 0.048 teeth per patient per year (95% CI: 0.033–0.096).

**Table 7:** Five-Year Tooth Survival and Cumulative Incidence Using Kaplan–Meier Analysis in Patients with Periodontal Disease.

Time (months)	Events (d)	Censored	At risk before (n)	Survival probability	Cumulative incidence
0	0	0	63	1.000	0.000
9	3	0	63	1 - 3/63 = 0.952	0.048
12	3	0	60	0.952 × (1 - 3/60) = 0.905	0.095
15	1	0	57	0.905 × (1 − 1/57) = 0.889	0.111
18	1	0	56	0.889 × (1 − 1/56) = 0.873	0.127
21	2	0	55	0.873 × (1 - 2/55) = 0.841	0.159
24	2	0	53	0.841 × (1 - 2/53) = 0.809	0.191
60	3	48	51	0.809 × (1 - 3/51) = 0.762	0.238

**Fig 2:**
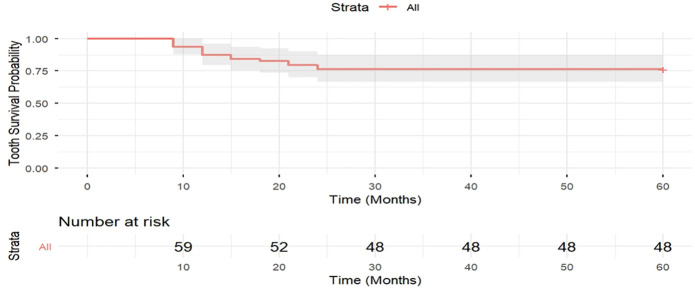
Kaplan–Meier survival curve for tooth loss. Fig 2 shows, overall probability of tooth survival over a 60-month period. The curve indicates a gradual decline in survival probability, with the number of teeth at risk decreasing over time.

**Fig 3:**
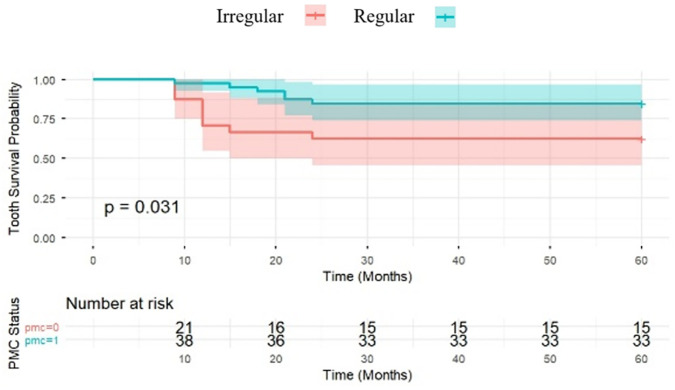
Kaplan–Meier survival curve for Periodontal maintenance Care (PMC). Fig 3 compares tooth survival between two categories of PMC. Patients who attended PMC regularly had higher tooth survival probability than those who were irregular for their visits. The difference between groups is statistically significant (p = 0.031), suggesting patients with PMC had positive impact on tooth retention.

**Fig 4:**
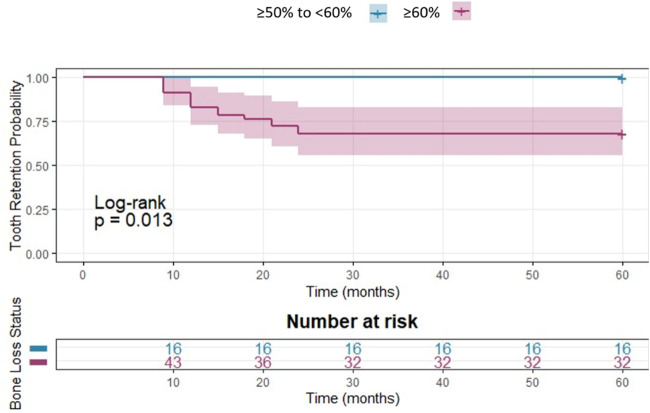
Kaplan–Meier survival curve for periodontal bone loss. Fig 4 examines tooth survival based on periodontal bone loss status. The survival curve demonstrates that baseline periodontal bone loss greater than >60% show a notably lower survival probability, i.e., higher risk of losing teeth over time compared to those with lower baseline bone loss, that showed increased likelihood of tooth retention. The log-rank test (p = 0.013) confirms a significant difference between the two categories.

The Kaplan–Meier survival curve for tooth loss, periodontal maintenance care and periodontal bone loss are depicted in Figs 2–4 respectively.

## Discussion

The objective of the present study was to evaluate tooth loss in young adults diagnosed with Grade C Periodontitis during periodontal maintenance care (PMC). Over a mean follow‑up period of 60 ± 2 months, tooth loss attributable to periodontal disease was low. Only 15 teeth (0.88%) were lost, and the majority of patients (76.19%) did not experience any tooth loss during the study period. When analyzed by tooth type, a slightly higher proportion of molar teeth (14.28%) were lost compared to non‑molars (9.52%), which is consistent with existing literature. Periodontal bone level less than 60%, MI-GC-P and periodontal maintenance care emerged as important factors associated with tooth retention. Survival analysis demonstrated that patients with severe baseline periodontal bone loss (>60%) had a markedly higher risk of tooth loss over time. Conversely, adherence to periodontal maintenance care showed a significant protective association, emphasizing the importance of regular maintenance care in preserving teeth.

Overall, the annual rate of tooth loss observed in this study (0.048 teeth per patient per year) was low and fell within the range reported in previous studies of patients receiving supportive periodontal therapy. This finding supports the concept that tooth loss during maintenance is often driven by a small subset of highly susceptible patients, rather than occurring uniformly across all individuals with Grade C Periodontitis [2,31]. Notably, longitudinal data on tooth loss during PMC in young adults with Grade C Periodontitis remain limited, particularly with respect to involvement of molars and incisors. The results of the present study therefore contribute meaningful data to this under‑represented patient population. The percentage of tooth loss reported in this study aligns with the majority of existing literature, which indicates a range of (0.01–0.24 teeth/patient/year) [9,32,33]. Patients receiving PMC are typically scheduled for recall appointments at three-month intervals during the initial year. This study further substantiates the efficacy of periodontal therapy for patients engaged in regular maintenance care in halting disease progression, thereby mitigating the rate of tooth loss [34,35].

In the subgroup, patients diagnosed with MI-GC-P retained significantly greater number of teeth in comparison to G-GC-P at both time intervals (APT, T1 and PMC, T2). Tooth loss rates were consistently lower in MI-GC-P cases compared to G-GC-P cases, suggesting that baseline disease extent influences long‑term outcomes following active periodontal therapy. [17]. The tooth loss was lower in MI-GC-P in comparison to G-GC-P. The comparatively favorable outcomes observed in localized disease may reflect a smaller number of affected teeth and a possible stabilization (“burn‑out”) of disease activity after initial rapid progression.[9]. The results are closest to study done by Amelie Bäumer in 2019 [32], with localized cases showing 0.014 teeth/patient/ year and generalized cases showing 0.118 teeth/patient/year of tooth loss. This is also comparable to multiple studies showing tooth loss in range between 0.01–0.24 for localized cases [15,36,37], and 0.05–0.31 [15,38,39], for generalized cases respectively.

Patients with baseline bone loss below 60% not only retained more teeth but also exhibited stability or improvement in bone levels during and at end of 5 years. In contrast, severe initial bone loss was associated with increased tooth loss risk, a finding that aligns with prior studies identifying baseline disease severity as a key predictive factor [9,32,33,40]. These results reinforce the importance of early diagnosis and intervention before extensive periodontal destruction occurs.

Compliance with periodontal maintenance care was strongly associated with tooth retention. Patients who attended regular maintenance visits lost fewer teeth and demonstrated a significant protective effect against tooth loss compared with those who were irregular. This observation agrees with previous studies showing that supportive periodontal care delivered at regular intervals can limit disease recurrence and reduce long‑term tooth loss, particularly in patients at higher periodontal risk [30]. The findings of the present study further emphasize that the success of periodontal therapy is highly dependent on sustained patient adherence to maintenance protocols [32,40–42].

Similar to previous reports, molars exhibited a higher tendency toward loss compared with non‑molars. [9,15]. This may be attributable to anatomical complexity, limited access for effective oral hygiene, and challenges associated with professional debridement in multirooted teeth [15,43]. No significant association was observed between smoking status or dental arch and tooth loss, which differs from some prior studies. This discrepancy may be explained by the relatively small number of active smokers, younger age of the cohort, shorter smoking exposure, and the structured emphasis on smoking cessation as part of the individualized treatment protocol [33,44]. Clinical periodontal parameters, including probing depths, clinical attachment levels showed stability or improvement at the 5‑year follow‑up. Furcation involvement and mobility were reduced following active therapy, supporting the effectiveness of non‑surgical periodontal treatment combined with consistent maintenance care [33,45].

Teeth initially categorized as having a hopeless prognosis were extracted during periodontal maintenance care, whereas teeth assessed as having a questionable prognosis were predominantly retained. All instances of tooth loss in the present study were attributable to periodontal causes, including advanced attachment loss, severe tooth mobility, and functional impairment related to supra‑eruption that adversely affected quality of life. These findings are in agreement with those reported by Graetz et al. in 2011 [33], who observed substantial survival of teeth with questionable and even hopeless prognosis following supportive periodontal therapy in patients with aggressive periodontitis.

Previous investigations have documented higher rates of periodontal‑related tooth loss during active therapy and supportive care among patients with generalized Stage IV or Grade C periodontitis [20,46]. In contrast, patients included in the present study demonstrated favorable clinical responses to non‑surgical periodontal therapy when coupled with regular maintenance care. Although severe periodontitis is associated with an increased risk of disease recurrence, existing evidence indicates that periodontal attachment loss can be stabilized following appropriate therapy, even in advanced disease stages [38].

In the context of increasing financial and resource constraints settings within healthcare systems, it is essential that periodontal stability achieved through specialist care is maintained over time. Prioritizing long‑term supportive periodontal therapy within treatment planning and healthcare policy is therefore crucial to maximizing tooth retention and optimizing long‑term patient outcomes. It is crucial to acknowledge that the treating dentist typically bears the responsibility for decisions regarding tooth extraction; thus, the objective should be to limit disease progression through sustained periodontal maintenance. Promoting adherence to maintenance care may reduce the likelihood of premature extractions or, at minimum, delay the removal of teeth with questionable or poor prognosis, particularly in younger patients [33]. The findings of this study should be interpreted considering certain limitations, including the retrospective design, the absence of radiographic recording at 5 years, and the potential influence of interaction effects. Nonetheless, the results highlight that, even in young adults with Grade C Periodontitis, periodontal stability and long‑term tooth retention are achievable when disease severity is addressed early and maintenance care is maintained.

## Conclusion

Tooth loss during periodontal maintenance care was minimal over the 5‑year follow‑up period. Severe periodontal bone loss (>60%) was significantly associated with an increased hazard of tooth loss, whereas adherence to periodontal maintenance care demonstrated a significant protective association. These findings underscore the importance of regular maintenance care in mitigating tooth loss and support the feasibility of long‑term retention of the majority of teeth through conservative periodontal management in patients diagnosed with Grade C-Periodontitis.

## Supporting information

S1 File Dataset. (XLSX)

## Abbreviations

APT: active periodontal therapy
CAL: clinical attachment loss
FI: furcation involvement
Grade C–MIP: Grade C molar‑incisor pattern
IRC: Institutional Review Committee
IOM: Institute of Medicine
PMC: periodontal maintenance care
PDs: probing depths
T.U.: Tribhuvan University
