# Supplementary material for: Tooth loss and associated factors during periodontal maintenance care in young adults with Grade C Periodontitis at a tertiary care center in Kathmandu, Nepal
Source: PLoS One. 2026 Jun 26;21(6):e0352255. doi: 10.1371/journal.pone.0352255 (PMC13309045; doi:10.1371/journal.pone.0352255)
Supplement: Attachment — Submitted filename: review.docx [file pone.0352255.s002.docx]

This manuscript evaluates 5-year tooth loss during periodontal maintenance care in 63 young patients diagnosed with grade C molar-incisor pattern periodontitis. The topic is clinically relevant and long-term follow-up data in this specific type are scarce. The authors report a low annual tooth loss rate (0.048 teeth/patient/year) and identify generalized disease and non-compliance as predictors of increased hazard of tooth loss.

While the clinical question is important and the follow-up duration is meaningful, there are substantial methodological and statistical limitations that weaken the strength of inference. The main concerns relate to model stability given the very low number of events, inconsistencies in unit of analysis (tooth vs patient), handling of predictors (particularly bone loss), and insufficient reporting of survival model assumptions.

Major revisions are required before the manuscript can be considered for publication.

The major issues are as follows:

1. Overfitting the cox model

The multivariate Cox model includes four predictors (gender, smoking, PMC compliance, disease extent) but only 15 events occurred.

This results in fewer than 4 events per variable (EPV ≈ 3.75), which is well below the recommended threshold (≈10 EPV) for stable estimation in Cox models. With such limited events	the hazard ratios are unstable, confidence intervals are really wide and effect sizes are inflated. The authors MUST justify the model strategy by either reducing the number of predictors, use penalized cox, represent the analysis as exploratory or report sensitivity analysis.

2. Exclusion of bone loss from the model

The manuscript states that bone loss was excluded because all 15 events occurred in patients with >60% bone loss.

This is not an appropriate reason to omit a predictor. Rather, this suggests complete or quasi-complete separation. In such cases a penalized regression model is justified and the predictive strength of severe bone loss should be modeled.

Given that bone loss appears to perfectly discriminate events, its exclusion likely biases the interpretation of other predictors.

3. Unit of analysis is inconsistent

The manuscript alternates between patient level analysis, tooth level descriptive stats, tooth level chi square comparisons and patient level comparison of mean numbers of teeth, however the survival analysis is clearly patient level, tooth level clustering is not modeled creating conceptual inconsistency so the authors should clearly define the primary unit of analysis, avoid mixing patient and tooth level inferences and clarify the denominators in table 4.

4. PMC compliance is used as a predictor of time to tooth loss raising potential of reverse causation and time dependent bias. The minimum here is that authors should define how compliance was operationalized, clarify whether it was assessed prospectively or retrospectively and acknowledge this limitation explicitly.
5. Proportional hazard assumptions were not reported

No report of testing the assumptions, shoenfeld residuals and log-log survival curves. Given the small number of events and apparent early separation of Kaplan–Meier curves, the PH assumption should be formally tested and reported.

6. Multiple testing without adjustment

Multiple subgroup comparisons are performed (independent t-tests, chi-square tests), yet no correction for multiple testing is mentioned. With small sample size and multiple comparisons, risk of type I error inflation exists. So authors should either apply adjustment, Bonferroni for example, or clearly state that the analysis is exploratory.

Moderate issues are as follows:

1. Smoking interpretation

The conclusion that smoking was not associated with tooth loss should be tempered. Only 17 patients were smokers. The study is likely underpowered to detect smoking effects. This should be acknowledged.

2. Effect size reporting

The manuscript emphasizes percentages and teeth per patient per year but does not report 5 year cumulative incidence and Kaplan-Meier survival probability at 60 months with 95% CI.

3. Table 4

The table titled “Association of tooth and patient characteristics with teeth loss” is unclear regarding whether the analysis is per patient or per tooth and what the denominator for percentages represents.

4. Calibration and measurement reliability

Although examiners were trained, no inter- or intra-examiner reliability statistics (kappa, ICC) are reported. This should be acknowledged as a limitation if not available.

Minor issues are as follows:

1. Some language in the discussion implies causality “regular follow-ups can significantly improve long-term tooth survival”. This should be softened to reflect association.
2. Minor grammatical corrections are needed throughout.
3. Provide number-at-risk table below the Kaplan-Meier curves.

Overall recommendation:

MAJOR REVISION

The study addresses an important clinical question and presents valuable long-term observational data. However, substantial methodological concerns particularly regarding the survival model stability, handling of predictors, and analytic consistency must be addressed before the conclusions can be considered reliable.

If the authors appropriately revise the statistical modeling and clarify analytical inconsistencies, the manuscript could make a meaningful contribution to the literature.
