# Supplementary material for: Tooth loss and associated factors during periodontal maintenance care in young adults with Grade C Periodontitis at a tertiary care center in Kathmandu, Nepal
Source: PLoS One. 2026 Jun 26;21(6):e0352255. doi: 10.1371/journal.pone.0352255 (PMC13309045; doi:10.1371/journal.pone.0352255)
Supplement: Attachment — Submitted filename: Response to reviewers.docx [file pone.0352255.s004.docx]

**Response to Reviewers	24.04.2026**

|  | Points raised by academic editor and reviewer(s) | Author comments |
| --- | --- | --- |
| 1 | TITLE "assessment of tooth loss," yet the results report only 15 teeth lost over a follow-up period of 60 ± 2 months. This outcome appears limited in magnitude relative to the strength of the title claim. I recommend revising the title to better reflect the scale and scope of the findings and to avoid potential overstatement. | The primary outcome variable/primary objective of this study was tooth loss, hence the term “assessment” and “tooth loss” was considered. The follow up was during their maintenance period of 5 years, which eventually showed loss of few teeth during periodontal treatment and maintenance. We have modified the title as “Tooth loss and associated factors during periodontal maintenance care in young adults with Grade C Periodontitis at a tertiary care center in Kathmandu, Nepal” |
| 2 | only 5 of the 37 cited sources (13.51%) were published within the last five years. literature base is not sufficiently up to date. Please revise and expand the reference list to include more recent and relevant studies, aiming for at least 50% of citations from the past five years where appropriate. | The literature reports scarcity of tooth loss in molar-incisor pattern periodontitis patients/aggressive periodontitis patients. Most literature available is based on chronic periodontitis patients. However, few references (recent) have been added to support the existing manuscript to best of author’s ability search in pubmed, scopus, web of science, google scholar. |
| 3 | no inter- or intra-examiner reliability data are presented. Calibration exercises, agreement statistics (such as kappa or ICC), and reproducibility measures should be reported. this point is critical and should be supported with objective data rather than general statements. | Noted. It was well performed at start of the study. It has been edited and added in the manuscript. |
| 4 | An essential clinical parameter is missing from the description of the disease presentation: the extent and distribution of periodontitis (localized versus generalized). Please update both the Materials & Methods and Results sections to include clear definitions and distributions of localized versus generalized involvement. | Definitions of localized versus generalized involvement have been added in material & method section. Periodontitis affecting young individuals (<35 years) presents different clinical characteristics and may encompass diverge presentations within the current classification system, even though they are grouped under the same denomination—Grade C Periodontitis. (Papapanou PN, Sanz M, et al. Periodontitis: Consensus report of Workgroup 2 of the 2017 World Workshop on the Classification of Periodontal and Peri-Implant Diseases and Conditions. J Clin Periodontol. 2018;45(Suppl 20):S162–S170). To ensure feasibility and avoid confusion, this study divided Grade C periodontitis based on clinical presentation of Nepalese patients to our OPD with molar-Incisor involvement in different degrees of severity, with Molar-incisor-Grade C Periodontitis (MI-GC-P), affecting at least first Molar and/or incisor, and less than 30% remaining teeth (maximum 7) and Generalized Grade C Periodontitis (G-GC-P) , when > 30% of remaining teeth affected (at least 8), affecting at least 3 permanent teeth other than affecting molars and/or incisors (Stolf CS, Paz HES, Paraluppi MC, et al. Molar-Incisor and Generalized Grade C Periodontitis: Distinct Microbiome-Immune Interactions Suggest Divergent Pathogenesis. *J Periodontal Res*. Published online February 10, 2026. doi:10.1111/jre.70077). The title of manuscript and nomenclature of localized and generalized have been modified and description added in manuscript with reference. |
| 5 | baseline prognostic classification criteria. Prognosis is highly relevant when interpreting tooth loss outcomes during maintenance and should be defined at the beginning of the study. In addition, incorporation of radiographic parameters would significantly strengthen the diagnostic characterization and risk assessment framework. | Baseline prognostic classification criteria have been added and modified in the manuscript. To best of author/s knowledge, it was more of a description of predictive factors which measured the factors/variables associated with response or lack of response (tooth loss risk- outcome) after specific periodontal therapy (APT in this study) (Clark, Gary M. “Prognostic factors versus predictive factors: Examples from a clinical trial of erlotinib.” *Molecular oncology* vol. 1,4 (2008): 406-12.)  Defining bone loss with baseline criteria less than or greater to 60 % was incorporated. The case was of stage III/IV periodontitis, less than 50% Bone loss was not considered in category of grade C. |
| 6 | the manuscript does not clearly demonstrate sufficient novelty to justify publication. The clinical question is known, and the present analysis does not appear to add a distinct methodological or conceptual contribution. The limitations in methodology and reporting further reduce the impact of the findings. Addressing the points above would substantially improve the scientific rigor and clarity of the work. | Thank you for the effective feedback. The above points have been added and the manuscript have been modified accordingly. |

Reviewer 2

| 1 | Overfitting the cox model. The multivariate Cox model includes four predictors (gender, smoking, PMC compliance, disease extent) but only 15 events occurred. | Due to limited number of events, and preventing overfitting of the cox model, we used AIC statistical model selection tool, we tried to penalize the predictors in the best model. Hence, the number of predictors were reduced, to represent the data which would be clinically relevant and statistically appropriate. The model (Multivariable Firth penalized Cox proportional hazards analysis) presented in the manuscript best represented the data. The overall model was statistically significant (likelihood ratio test p = 0.0016), indicating good explanatory ability. |
| --- | --- | --- |
| 2 | This results in fewer than 4 events per variable (EPV ≈ 3.75), which is well below the recommended threshold (≈10 EPV) for stable estimation in Cox models. With such limited events the hazard ratios are unstable, confidence intervals are really wide and effect sizes are inflated. The authors MUST justify the model strategy by either reducing the number of predictors, use penalized cox, represent the analysis as exploratory or report sensitivity analysis. |  |
| 3 | The manuscript states that bone loss was excluded because all 15 events occurred in patients with >60% bone loss. This is not an appropriate reason to omit a predictor. Rather, this suggests complete or quasi-complete separation. In such cases a penalized regression model is justified and the predictive strength of severe bone loss should be modeled. Given that bone loss appears to perfectly discriminate events, its exclusion likely biases the interpretation of other predictors. | We have used penalized regression model and included in the manuscript. |
| 4 | The manuscript alternates between patient level analysis, tooth level descriptive stats, tooth level chi square comparisons and patient level comparison of mean numbers of teeth, however the survival analysis is clearly patient level, tooth level clustering is not modeled creating conceptual inconsistency so the authors should clearly define the primary unit of analysis, avoid mixing patient and tooth level inferences and clarify the denominators in table 4. | Denominators in table 4 is 63 which mean 63 patients, it is patient level analysis. Only table 2 presents tooth level descriptive analysis. Table 4 is now edited for better understanding for readers. |
| 5 | PMC compliance is used as a predictor of time to tooth loss raising potential of reverse causation and time dependent bias. The minimum here is that authors should define how compliance was operationalized, clarify whether it was assessed prospectively or retrospectively and acknowledge this limitation explicitly. | Regular compliers were those who visited for PMC quarterly during first year and subsequently annually/biannually in ensuring years. Irregular compliers were mostly who visited us, but failed to visit quarterly during first year. (Seirafi AH, Ebrahimi R, Golkari A, Khosropanah H, Soolari A. Tooth loss assessment during periodontal maintenance in erratic versus complete compliance in a periodontal private practice in Shiraz, Iran: a 10-year retrospective study. *J Int Acad Periodontol*. 2014;16(2):43-49.).  PMC was assessed prospectively. |
| 6 | Proportional hazard assumptions were not reported No report of testing the assumptions, shoenfeld residuals and log-log survival curves. Given the small number of events and apparent early separation of Kaplan–Meier curves, the PH assumption should be formally tested and reported. | It is reported in the manuscript |
|  |  |  |

| 1. | 1.Smoking interpretation The conclusion that smoking was not associated with tooth loss should be tempered. Only 17 patients were smokers. The study is likely underpowered to detect smoking effects. This should be acknowledged. | Smoking is a significant risk factors of periodontitis. The number of patients and the duration of active smokers of patients in this study was <3 years which probably did not show a significant effect on periodontium. The reasons attributed to it were added in the discussion. Also, now a reference has been added to justify the study findings and further referral for readers. |
| --- | --- | --- |
| 2 | Effect size reporting The manuscript emphasizes percentages and teeth per patient per year but does not report 5 year cumulative incidence and Kaplan-Meier survival probability at 60 months with 95% CI. | Reported in the manuscript (table 7) |
| 3 | The table titled “Association of tooth and patient characteristics with teeth loss” is unclear regarding whether the analysis is per patient or per tooth and what the denominator for percentages represents. | The denominator percentages are individual patients, not tooth. The table title has been modified for better understanding |
| 4 | Calibration and measurement reliability Although examiners were trained, no inter- or intra-examiner reliability statistics (kappa, ICC) are reported. This should be acknowledged as a limitation if not available. | Noted. It was well performed at start of the study. It has been edited and added in the manuscript |
| 5 | Some language in the discussion implies causality “regular follow-ups can significantly improve long-term tooth survival”. This should be softened to reflect association. | The language has been modified. |
| 6 | 2.Minor grammatical corrections are needed throughout. | Checked and corrections made to best of authors ability |
| 7 | Provide number-at-risk table below the Kaplan-Meier curves. | Provided |

Reviewer 3

| 1 | Please move the whole paragraph of Statistical analysis at the end of the Methods section. In the statistical analysis part please refer e.g. using a stat. book(s) the methods used e.g. t-test, rank-sum test etc. Also please add with reference a test of normalty of the data because you should have tested it since you are publishing mean and SD and median and IQR. You are mentioning the mean age and SD of your patient, if it possible you can show your results in age groups as well e.g. two age groups at T1,T2. | Kolmogorov-Smirnov (K-S) test was used for checking the normality distribution of data. Parametric tests were used for variables showing normal distribution and non-parametric tests for those showing skewed distribution.  The age groups were recoded at completion of APT (T1), and hence at T2 (end of 5 year PMC). |
| --- | --- | --- |
| 2 | . In Table 2 Tooth lost is presented as 07 etc. please just show these numbres as integers. | Edited |
| 3 | On Table 2 please explain what is PMC. In Table 3 & Table 4 please write out what is PMC again. | Mentioned in the table. |
| 4 | On Table 5 please write out what is PD and CAL. | Mentioned in the table. |
| 5 | Table 6 please show only Hazard Ratio, 95% CI and p-value in this order. | Done |
| 6 | Please explain what is PMC here. | Mentioned at the bottom of table |
| 7 | On Fig 2 to Fig 5 please show the 95% confidence intervals for both hazard and survival functions. | Explained though figure |
| 8 | You are using abbreviations all along i your manuscript, please define just once e.g. PD, CAL are written out twice, please check out these. | Edited. mentioned only once |
| 9 | "As seen in other studies, baseline diagnosis (Baumer, El Sayed, et al., 2011a) had an impact on tooth loss and periodontal destruction after APT." in the Discussion is in different letter type and refrence also doesn't fit with reference style of Plos One, please correct it. | Sure. It is edited and amended. |
| 10 | In the Discussion when you summarise your findng mentioned the age of the patients. There are no page numbers, please add them. | Sure. Edited in the manuscript. |
