# Supplementary material for: Tooth loss and associated factors during periodontal maintenance care in young adults with Grade C Periodontitis at a tertiary care center in Kathmandu, Nepal
Source: PLoS One. 2026 Jun 26;21(6):e0352255. doi: 10.1371/journal.pone.0352255 (PMC13309045; doi:10.1371/journal.pone.0352255)
Supplement: Attachment — Submitted filename: response_to_reviewers_auresp_2.docx [file pone.0352255.s005.docx]

Response to Reviewers

|  | Journal Requirements | Response |
| --- | --- | --- |
| 1 | If the reviewer comments include a recommendation to cite specific previously published works, please review and evaluate these publications to determine whether they are relevant and should be cited. There is no requirement to cite these works unless the editor has indicated otherwise. | All the references seem justified with the manuscript. |
| 2. | Please review your reference list to ensure that it is complete and correct. If you have cited papers that have been retracted, please include the rationale for doing so in the manuscript text, or remove these references and replace them with relevant current references. Any changes to the reference list should be mentioned in the rebuttal letter that accompanies your revised manuscript. If you need to cite a retracted article, indicate the article’s retracted status in the References list and also include a citation and full reference for the retraction notice. | Reviewed all the references. No changes in the current references. All the references have been checked and edited. |
|  | Editor’s & reviewer’s comments |  |
| 3. | Please move the "Statistical analysis" from "Results" to as last section of "Methods", as Reviewer 3 commented previously. The references of "Statistical analysis" used don't match the PlosOne refrence style and the rest of the manuscript. Please correct it. | Edited. |
