# Supplementary material for: Tooth loss and associated factors during periodontal maintenance care in young adults with Grade C Periodontitis at a tertiary care center in Kathmandu, Nepal
Source: PLoS One. 2026 Jun 26;21(6):e0352255. doi: 10.1371/journal.pone.0352255 (PMC13309045; doi:10.1371/journal.pone.0352255)
Supplement: Peer review history

# Peer review: Decision Letter 0

**DOI:** 10.1371/journal.pone.0352255.r001

**Authors:** Attila Csaba Nagy

10 Mar 2026

PONE-D-26-00010 Assessment of tooth loss during periodontal maintenance in grade C molar-incisor pattern periodontitis patients in a tertiary care center of Kathmandu, Nepal PLOS One

Dear Dr. Goel,

Thank you for submitting your manuscript to PLOS ONE. After careful consideration, we feel that it has merit but does not fully meet PLOS ONE’s publication criteria as it currently stands. Therefore, we invite you to submit a revised version of the manuscript that addresses the points raised during the review process.

Please submit your revised manuscript by Apr 24 2026 11:59PM. If you will need more time than this to complete your revisions, please reply to this message or contact the journal office at plosone@plos.org. When you're ready to submit your revision, log on to https://www.editorialmanager.com/pone/ and select the 'Submissions Needing Revision' folder to locate your manuscript file.

Please include the following items when submitting your revised manuscript:

- A letter that responds to each point raised by the academic editor and reviewer(s). You should upload this letter as a separate file labeled 'Response to Reviewers'.
- A marked-up copy of your manuscript that highlights changes made to the original version. You should upload this as a separate file labeled 'Revised Manuscript with Track Changes'.
- An unmarked version of your revised paper without tracked changes. You should upload this as a separate file labeled 'Manuscript'.

If you would like to make changes to your financial disclosure, please include your updated statement in your cover letter. Guidelines for resubmitting your figure files are available below the reviewer comments at the end of this letter.

If applicable, we recommend that you deposit your laboratory protocols in protocols.io to enhance the reproducibility of your results. Protocols.io assigns your protocol its own identifier (DOI) so that it can be cited independently in the future. For instructions see: https://journals.plos.org/plosone/s/submission-guidelines#loc-laboratory-protocols. Additionally, PLOS ONE offers an option for publishing peer-reviewed Lab Protocol articles, which describe protocols hosted on protocols.io. Read more information on sharing protocols at https://plos.org/protocols?utm_medium=editorial-email&utm_source=authorletters&utm_campaign=protocols.

We look forward to receiving your revised manuscript.

Kind regards,

Attila Csaba Nagy

Academic Editor

PLOS One

Journal Requirements:

When submitting your revision, we need you to address these additional requirements.

1. Please ensure that your manuscript meets PLOS ONE's style requirements, including those for file naming. The PLOS ONE style templates can be found at

https://journals.plos.org/plosone/s/file?id=wjVg/PLOSOne_formatting_sample_main_body.pdf and https://journals.plos.org/plosone/s/file?id=ba62/PLOSOne_formatting_sample_title_authors_affiliations.pdf

2. When completing the data availability statement of the submission form, you indicated that you will make your data available on acceptance. We strongly recommend all authors decide on a data sharing plan before acceptance, as the process can be lengthy and hold up publication timelines. Please note that, though access restrictions are acceptable now, your entire data will need to be made freely accessible if your manuscript is accepted for publication. This policy applies to all data except where public deposition would breach compliance with the protocol approved by your research ethics board. If you are unable to adhere to our open data policy, please kindly revise your statement to explain your reasoning and we will seek the editor's input on an exemption. Please be assured that, once you have provided your new statement, the assessment of your exemption will not hold up the peer review process.

3. Please include a separate caption for each figure in your manuscript.

4. Please include your tables as part of your main manuscript and remove the individual files. Please note that supplementary tables should remain as separate "Supporting Information" files.

5. Please include captions for your Supporting Information files at the end of your manuscript, and update any in-text citations to match accordingly. Please see our Supporting Information guidelines for more information: http://journals.plos.org/plosone/s/supporting-information.

If the reviewer comments include a recommendation to cite specific previously published works, please review and evaluate these publications to determine whether they are relevant and should be cited. There is no requirement to cite these works unless the editor has indicated otherwise.

[Note: HTML markup is below. Please do not edit.]

Reviewers' comments:

Reviewer's Responses to Questions

**Comments to the Author**

1. Is the manuscript technically sound, and do the data support the conclusions?

The manuscript must describe a technically sound piece of scientific research with data that supports the conclusions. Experiments must have been conducted rigorously, with appropriate controls, replication, and sample sizes. The conclusions must be drawn appropriately based on the data presented.

Reviewer #1: Yes

Reviewer #2: Partly

Reviewer #3: Yes

**********

2. Has the statistical analysis been performed appropriately and rigorously?

Reviewer #1: No

Reviewer #2: No

Reviewer #3: Yes

**********

3. Have the authors made all data underlying the findings in their manuscript fully available?

The PLOS Data policy requires authors to make all data underlying the findings described in their manuscript fully available without restriction, with rare exception (please refer to the Data Availability Statement in the manuscript PDF file). The data should be provided as part of the manuscript or its supporting information, or deposited to a public repository. For example, in addition to summary statistics, the data points behind means, medians and variance measures should be available. If there are restrictions on publicly sharing data—e.g. participant privacy or use of data from a third party—those must be specified.

Reviewer #1: Yes

Reviewer #2: Yes

Reviewer #3: Yes

**********

4. Is the manuscript presented in an intelligible fashion and written in standard English?

PLOS ONE does not copyedit accepted manuscripts, so the language in submitted articles must be clear, correct, and unambiguous. Any typographical or grammatical errors should be corrected at revision, so please note any specific errors here.

Reviewer #1: Yes

Reviewer #2: Yes

Reviewer #3: Yes

**********

5. Review Comments to the Author

Please use the space provided to explain your answers to the questions above. You may also include additional comments for the author, including concerns about dual publication, research ethics, or publication ethics. (Please upload your review as an attachment if it exceeds 20,000 characters)

Reviewer #1: Dear Authors,

I have carefully evaluated the manuscript entitled "Assessment of tooth loss during periodontal maintenance in grade C molar-incisor pattern periodontitis patients in a tertiary care center of Kathmandu, Nepal." The stated objective of the study is to assess tooth loss during periodontal maintenance in patients with grade C molar-incisor pattern periodontitis (grade C-MIP) and to identify predictors associated with tooth-loss risk. The topic is clinically relevant; however, several conceptual and methodological aspects require clarification and strengthening before the work can be considered for publication.

Regarding the TITLE, the current phrasing emphasizes an "assessment of tooth loss," yet the results report only 15 teeth lost over a follow-up period of 60 ± 2 months. This outcome appears limited in magnitude relative to the strength of the title claim. I recommend revising the title to better reflect the scale and scope of the findings and to avoid potential overstatement.

Concerning the REFERENCES, only 5 of the 37 cited sources (13.51%) were published within the last five years. This suggests that the literature base is not sufficiently up to date. Please revise and expand the reference list to include more recent and relevant studies, aiming for at least 50% of citations from the past five years where appropriate.

In the MATERIALS & METHODS and RESULTS sections, examiner calibration requires clearer documentation. The manuscript states that "the periodontal examination was conducted by dental hygienists supported by an experienced periodontist," and that dental hygienists are well trained for periodontal calibrations. However, no inter- or intra-examiner reliability data are presented. Calibration exercises, agreement statistics (such as kappa or ICC), and reproducibility measures should be reported. Given known variability in periodontal probing and diagnosis across clinicians with different backgrounds and training, this point is critical and should be supported with objective data rather than general statements.

An essential clinical parameter is missing from the description of the disease presentation: the extent and distribution of periodontitis (localized versus generalized). This distinction has major prognostic and therapeutic implications, especially in grade C molar-incisor pattern cases. Please update both the Materials & Methods and Results sections to include clear definitions and distributions of localized versus generalized involvement.

The study would also benefit from including baseline prognostic classification criteria. Prognosis is highly relevant when interpreting tooth loss outcomes during maintenance and should be defined at the beginning of the study. In addition, incorporation of radiographic parameters would significantly strengthen the diagnostic characterization and risk assessment framework.

Finally, in its current form, the manuscript does not clearly demonstrate sufficient novelty to justify publication. The clinical question is known, and the present analysis does not appear to add a distinct methodological or conceptual contribution. The limitations in methodology and reporting further reduce the impact of the findings. Addressing the points above would substantially improve the scientific rigor and clarity of the work.

Reviewer #2: This manuscript evaluates 5-year tooth loss during periodontal maintenance care in 63 young patients diagnosed with grade C molar-incisor pattern periodontitis. The topic is clinically relevant and long-term follow-up data in this specific type are scarce. The authors report a low annual tooth loss rate (0.048 teeth/patient/year) and identify generalized disease and non-compliance as predictors of increased hazard of tooth loss.

While the clinical question is important and the follow-up duration is meaningful, there are substantial methodological and statistical limitations that weaken the strength of inference. The main concerns relate to model stability given the very low number of events, inconsistencies in unit of analysis (tooth vs patient), handling of predictors (particularly bone loss), and insufficient reporting of survival model assumptions.

Major revisions are required before the manuscript can be considered for publication.

The major issues are as follows:

1.Overfitting the cox model

The multivariate Cox model includes four predictors (gender, smoking, PMC compliance, disease extent) but only 15 events occurred.

This results in fewer than 4 events per variable (EPV ≈ 3.75), which is well below the recommended threshold (≈10 EPV) for stable estimation in Cox models. With such limited events the hazard ratios are unstable, confidence intervals are really wide and effect sizes are inflated. The authors MUST justify the model strategy by either reducing the number of predictors, use penalized cox, represent the analysis as exploratory or report sensitivity analysis.

2.Exclusion of bone loss from the model

The manuscript states that bone loss was excluded because all 15 events occurred in patients with >60% bone loss.

This is not an appropriate reason to omit a predictor. Rather, this suggests complete or quasi-complete separation. In such cases a penalized regression model is justified and the predictive strength of severe bone loss should be modeled.

Given that bone loss appears to perfectly discriminate events, its exclusion likely biases the interpretation of other predictors.

3.Unit of analysis is inconsistent

The manuscript alternates between patient level analysis, tooth level descriptive stats, tooth level chi square comparisons and patient level comparison of mean numbers of teeth, however the survival analysis is clearly patient level, tooth level clustering is not modeled creating conceptual inconsistency so the authors should clearly define the primary unit of analysis, avoid mixing patient and tooth level inferences and clarify the denominators in table 4.

4.PMC compliance is used as a predictor of time to tooth loss raising potential of reverse causation and time dependent bias. The minimum here is that authors should define how compliance was operationalized, clarify whether it was assessed prospectively or retrospectively and acknowledge this limitation explicitly.

5.Proportional hazard assumptions were not reported

No report of testing the assumptions, shoenfeld residuals and log-log survival curves. Given the small number of events and apparent early separation of Kaplan–Meier curves, the PH assumption should be formally tested and reported.

6.Multiple testing without adjustment

Multiple subgroup comparisons are performed (independent t-tests, chi-square tests), yet no correction for multiple testing is mentioned. With small sample size and multiple comparisons, risk of type I error inflation exists. So authors should either apply adjustment, Bonferroni for example, or clearly state that the analysis is exploratory.

Moderate issues are as follows:

1.Smoking interpretation

The conclusion that smoking was not associated with tooth loss should be tempered. Only 17 patients were smokers. The study is likely underpowered to detect smoking effects. This should be acknowledged.

2.Effect size reporting

The manuscript emphasizes percentages and teeth per patient per year but does not report 5 year cumulative incidence and Kaplan-Meier survival probability at 60 months with 95% CI.

3.Table 4

The table titled “Association of tooth and patient characteristics with teeth loss” is unclear regarding whether the analysis is per patient or per tooth and what the denominator for percentages represents.

4.Calibration and measurement reliability

Although examiners were trained, no inter- or intra-examiner reliability statistics (kappa, ICC) are reported. This should be acknowledged as a limitation if not available.

Minor issues are as follows:

1.Some language in the discussion implies causality “regular follow-ups can significantly improve long-term tooth survival”. This should be softened to reflect association.

2.Minor grammatical corrections are needed throughout.

3.Provide number-at-risk table below the Kaplan-Meier curves.

Overall recommendation:

MAJOR REVISION

The study addresses an important clinical question and presents valuable long-term observational data. However, substantial methodological concerns particularly regarding the survival model stability, handling of predictors, and analytic consistency must be addressed before the conclusions can be considered reliable.

If the authors appropriately revise the statistical modeling and clarify analytical inconsistencies, the manuscript could make a meaningful contribution to the literature.

Reviewer #3: Dear Authors,

This is a well written manuscript, especially the Discussion, however it requires a major review:

Please move the whole paragraph of Statistical analysis at the end of the Methods section. In the statistical analysis part please refer e.g. using a stat. book(s) the methods used e.g. t-test, rank-sum test etc. Also please add with reference a test of normalty of the data because you should have tested it since you are publishing mean and SD and median and IQR. You are mentioning the mean age and SD of your patient, if it possible you can show your results in age groups as well e.g. two age groups at T1,T2. Some specific comments . In Table 2 Tooth lost is presented as 07 etc. please just show these numbres as integers. On Table 2 please explain what is PMC. In Table 3 & Table 4 please write out what is PMC again. On Table 5 please write out what is PD and CAL. Table 6 please show only Hazard Ratio, 95% CI and p-value in this order. Please explain what is PMC here. On Fig 2 to Fig 5 please show the 95% confidence intervals for both hazard and survival functions.

You are using abbreviations all along i your manuscript, please define just once e.g. PD,CAL are written out twice, please check out these.

"As seen in other studies, baseline diagnosis (Baumer, El Sayed, et al., 2011a) had an impact on tooth loss

and periodontal destruction after APT." in the Discussion is in different letter type and refrence also doesn't fit with reference style of Plos One, please correct it.

In the Discussion when you summarise your findng mentioned the age of the patients.

There are no page numbers, please add them.

Yours sincerely.

**********

6. PLOS authors have the option to publish the peer review history of their article (what does this mean?). If published, this will include your full peer review and any attached files.

If you choose “no”, your identity will remain anonymous but your review may still be made public.

**Do you want your identity to be public for this peer review?** For information about this choice, including consent withdrawal, please see our Privacy Policy.

Reviewer #1: No

Reviewer #2: No

Reviewer #3: No

**********

[NOTE: If reviewer comments were submitted as an attachment file, they will be attached to this email and accessible via the submission site. Please log into your account, locate the manuscript record, and check for the action link "View Attachments". If this link does not appear, there are no attachment files.]

To ensure your figures meet our technical requirements, please review our figure guidelines: https://journals.plos.org/plosone/s/figures

You may also use PLOS’s free figure tool, NAAS, to help you prepare publication quality figures: https://journals.plos.org/plosone/s/figures#loc-tools-for-figure-preparation.

NAAS will assess whether your figures meet our technical requirements by comparing each figure against our figure specifications.

Attachment Submitted filename: review.docx

# Author comment: Author response to Decision Letter 1

**DOI:** 10.1371/journal.pone.0352255.r002

2 May 2026

Response to Reviewers 24.04.2026

Points raised by academic editor and reviewer(s) Author comments

1 TITLE "assessment of tooth loss," yet the results report only 15 teeth lost over a follow-up period of 60 ± 2 months. This outcome appears limited in magnitude relative to the strength of the title claim. I recommend revising the title to better reflect the scale and scope of the findings and to avoid potential overstatement.

The primary outcome variable/primary objective of this study was tooth loss, hence the term “assessment” and “tooth loss” was considered. The follow up was during their maintenance period of 5 years, which eventually showed loss of few teeth during periodontal treatment and maintenance. We have modified the title as “Tooth loss and associated factors during periodontal maintenance care in young adults with Grade C Periodontitis at a tertiary care center in Kathmandu, Nepal”

2 only 5 of the 37 cited sources (13.51%) were published within the last five years. literature base is not sufficiently up to date. Please revise and expand the reference list to include more recent and relevant studies, aiming for at least 50% of citations from the past five years where appropriate. The literature reports scarcity of tooth loss in molar-incisor pattern periodontitis patients/aggressive periodontitis patients. Most literature available is based on chronic periodontitis patients. However, few references (recent) have been added to support the existing manuscript to best of author’s ability search in pubmed, scopus, web of science, google scholar.

3 no inter- or intra-examiner reliability data are presented. Calibration exercises, agreement statistics (such as kappa or ICC), and reproducibility measures should be reported. this point is critical and should be supported with objective data rather than general statements.

Noted. It was well performed at start of the study. It has been edited and added in the manuscript.

4 An essential clinical parameter is missing from the description of the disease presentation: the extent and distribution of periodontitis (localized versus generalized). Please update both the Materials & Methods and Results sections to include clear definitions and distributions of localized versus generalized involvement.

Definitions of localized versus generalized involvement have been added in material & method section. Periodontitis affecting young individuals (<35 years) presents different clinical characteristics and may encompass diverge presentations within the current classification system, even though they are grouped under the same denomination—Grade C Periodontitis. (Papapanou PN, Sanz M, et al. Periodontitis: Consensus report of Workgroup 2 of the 2017 World Workshop on the Classification of Periodontal and Peri-Implant Diseases and Conditions. J Clin Periodontol. 2018;45(Suppl 20):S162–S170). To ensure feasibility and avoid confusion, this study divided Grade C periodontitis based on clinical presentation of Nepalese patients to our OPD with molar-Incisor involvement in different degrees of severity, with Molar-incisor-Grade C Periodontitis (MI-GC-P), affecting at least first Molar and/or incisor, and less than 30% remaining teeth (maximum 7) and Generalized Grade C Periodontitis (G-GC-P) , when > 30% of remaining teeth affected (at least 8), affecting at least 3 permanent teeth other than affecting molars and/or incisors (Stolf CS, Paz HES, Paraluppi MC, et al. Molar-Incisor and Generalized Grade C Periodontitis: Distinct Microbiome-Immune Interactions Suggest Divergent Pathogenesis. J Periodontal Res. Published online February 10, 2026. doi:10.1111/jre.70077). The title of manuscript and nomenclature of localized and generalized have been modified and description added in manuscript with reference.

5 baseline prognostic classification criteria. Prognosis is highly relevant when interpreting tooth loss outcomes during maintenance and should be defined at the beginning of the study. In addition, incorporation of radiographic parameters would significantly strengthen the diagnostic characterization and risk assessment framework.

Baseline prognostic classification criteria have been added and modified in the manuscript. To best of author/s knowledge, it was more of a description of predictive factors which measured the factors/variables associated with response or lack of response (tooth loss risk- outcome) after specific periodontal therapy (APT in this study) (Clark, Gary M. “Prognostic factors versus predictive factors: Examples from a clinical trial of erlotinib.” Molecular oncology vol. 1,4 (2008): 406-12.)

Defining bone loss with baseline criteria less than or greater to 60 % was incorporated. The case was of stage III/IV periodontitis, less than 50% Bone loss was not considered in category of grade C.

6 the manuscript does not clearly demonstrate sufficient novelty to justify publication. The clinical question is known, and the present analysis does not appear to add a distinct methodological or conceptual contribution. The limitations in methodology and reporting further reduce the impact of the findings. Addressing the points above would substantially improve the scientific rigor and clarity of the work.

Thank you for the effective feedback. The above points have been added and the manuscript have been modified accordingly.

Reviewer 2

1 Overfitting the cox model. The multivariate Cox model includes four predictors (gender, smoking, PMC compliance, disease extent) but only 15 events occurred. Due to limited number of events, and preventing overfitting of the cox model, we used AIC statistical model selection tool, we tried to penalize the predictors in the best model. Hence, the number of predictors were reduced, to represent the data which would be clinically relevant and statistically appropriate. The model (Multivariable Firth penalized Cox proportional hazards analysis) presented in the manuscript best represented the data. The overall model was statistically significant (likelihood ratio test p = 0.0016), indicating good explanatory ability.

2 This results in fewer than 4 events per variable (EPV ≈ 3.75), which is well below the recommended threshold (≈10 EPV) for stable estimation in Cox models. With such limited events the hazard ratios are unstable, confidence intervals are really wide and effect sizes are inflated. The authors MUST justify the model strategy by either reducing the number of predictors, use penalized cox, represent the analysis as exploratory or report sensitivity analysis.

3 The manuscript states that bone loss was excluded because all 15 events occurred in patients with >60% bone loss.

This is not an appropriate reason to omit a predictor. Rather, this suggests complete or quasi-complete separation. In such cases a penalized regression model is justified and the predictive strength of severe bone loss should be modeled.

Given that bone loss appears to perfectly discriminate events, its exclusion likely biases the interpretation of other predictors.

We have used penalized regression model and included in the manuscript.

4 The manuscript alternates between patient level analysis, tooth level descriptive stats, tooth level chi square comparisons and patient level comparison of mean numbers of teeth, however the survival analysis is clearly patient level, tooth level clustering is not modeled creating conceptual inconsistency so the authors should clearly define the primary unit of analysis, avoid mixing patient and tooth level inferences and clarify the denominators in table 4. Denominators in table 4 is 63 which mean 63 patients, it is patient level analysis. Only table 2 presents tooth level descriptive analysis. Table 4 is now edited for better understanding for readers.

5 PMC compliance is used as a predictor of time to tooth loss raising potential of reverse causation and time dependent bias. The minimum here is that authors should define how compliance was operationalized, clarify whether it was assessed prospectively or retrospectively and acknowledge this limitation explicitly. Regular compliers were those who visited for PMC quarterly during first year and subsequently annually/biannually in ensuring years. Irregular compliers were mostly who visited us, but failed to visit quarterly during first year. (Seirafi AH, Ebrahimi R, Golkari A, Khosropanah H, Soolari A. Tooth loss assessment during periodontal maintenance in erratic versus complete compliance in a periodontal private practice in Shiraz, Iran: a 10-year retrospective study. J Int Acad Periodontol. 2014;16(2):43-49.).

PMC was assessed prospectively.

6 Proportional hazard assumptions were not reported

No report of testing the assumptions, shoenfeld residuals and log-log survival curves. Given the small number of events and apparent early separation of Kaplan–Meier curves, the PH assumption should be formally tested and reported. It is reported in the manuscript

1. 1.Smoking interpretation

The conclusion that smoking was not associated with tooth loss should be tempered. Only 17 patients were smokers. The study is likely underpowered to detect smoking effects. This should be acknowledged.

Smoking is a significant risk factors of periodontitis. The number of patients and the duration of active smokers of patients in this study was <3 years which probably did not show a significant effect on periodontium. The reasons attributed to it were added in the discussion. Also, now a reference has been added to justify the study findings and further referral for readers.

2 Effect size reporting

The manuscript emphasizes percentages and teeth per patient per year but does not report 5 year cumulative incidence and Kaplan-Meier survival probability at 60 months with 95% CI.

Reported in the manuscript (table 7)

3 The table titled “Association of tooth and patient characteristics with teeth loss” is unclear regarding whether the analysis is per patient or per tooth and what the denominator for percentages represents. The denominator percentages are individual patients, not tooth. The table title has been modified for better understanding

4 Calibration and measurement reliability

Although examiners were trained, no inter- or intra-examiner reliability statistics (kappa, ICC) are reported. This should be acknowledged as a limitation if not available.

Noted. It was well performed at start of the study. It has been edited and added in the manuscript

5 Some language in the discussion implies causality “regular follow-ups can significantly improve long-term tooth survival”. This should be softened to reflect association.

The language has been modified.

6 2.Minor grammatical corrections are needed throughout.

Checked and corrections made to best of authors ability

7 Provide number-at-risk table below the Kaplan-Meier curves.

Provided

Reviewer 3

1 Please move the whole paragraph of Statistical analysis at the end of the Methods section. In the statistical analysis part please refer e.g. using a stat. book(s) the methods used e.g. t-test, rank-sum test etc. Also please add with reference a test of normalty of the data because you should have tested it since you are publishing mean and SD and median and IQR. You are mentioning the mean age and SD of your patient, if it possible you can show your results in age groups as well e.g. two age groups at T1,T2. Kolmogorov-Smirnov (K-S) test was used for checking the normality distribution of data. Parametric tests were used for variables showing normal distribution and non-parametric tests for those showing skewed distribution.

The age groups were recoded at completion of APT (T1), and hence at T2 (end of 5 year PMC).

2 . In Table 2 Tooth lost is presented as 07 etc. please just show these numbres as integers. Edited

3 On Table 2 please explain what is PMC. In Table 3 & Table 4 please write out what is PMC again. Mentioned in the table.

4 On Table 5 please write out what is PD and CAL. Mentioned in the table.

5 Table 6 please show only Hazard Ratio, 95% CI and p-value in this order. Done

6 Please explain what is PMC here. Mentioned at the bottom of table

7 On Fig 2 to Fig 5 please show the 95% confidence intervals for both hazard and survival functions.

Explained though figure

8 You are using abbreviations all along i your manuscript, please define just once e.g. PD, CAL are written out twice, please check out these. Edited. mentioned only once

9 "As seen in other studies, baseline diagnosis (Baumer, El Sayed, et al., 2011a) had an impact on tooth loss

and periodontal destruction after APT." in the Discussion is in different letter type and refrence also doesn't fit with reference style of Plos One, please correct it. Sure. It is edited and amended.

10 In the Discussion when you summarise your findng mentioned the age of the patients.

There are no page numbers, please add them.

Sure. Edited in the manuscript.

Attachment Submitted filename: Response to reviewers.docx

# Peer review: Decision Letter 1

**DOI:** 10.1371/journal.pone.0352255.r003

**Authors:** Attila Csaba Nagy, Attila Csaba Nagy

17 May 2026

PONE-D-26-00010R1 Tooth loss and associated factors during periodontal maintenance care in young adults with Grade C Periodontitis at a tertiary care center in Kathmandu, Nepal PLOS One

Dear Dr. Goel,

Thank you for submitting your manuscript to PLOS ONE. After careful consideration, we feel that it has merit but does not fully meet PLOS ONE’s publication criteria as it currently stands. Therefore, we invite you to submit a revised version of the manuscript that addresses the points raised during the review process.

Please submit your revised manuscript by Jul 01 2026 11:59PM. If you will need more time than this to complete your revisions, please reply to this message or contact the journal office at plosone@plos.org. When you're ready to submit your revision, log on to https://www.editorialmanager.com/pone/ and select the 'Submissions Needing Revision' folder to locate your manuscript file.

Please include the following items when submitting your revised manuscript:

- A letter that responds to each point raised by the academic editor and reviewer(s). You should upload this letter as a separate file labeled 'Response to Reviewers'.
- A marked-up copy of your manuscript that highlights changes made to the original version. You should upload this as a separate file labeled 'Revised Manuscript with Track Changes'.
- An unmarked version of your revised paper without tracked changes. You should upload this as a separate file labeled 'Manuscript'.

If you would like to make changes to your financial disclosure, please include your updated statement in your cover letter. Guidelines for resubmitting your figure files are available below the reviewer comments at the end of this letter.

If applicable, we recommend that you deposit your laboratory protocols in protocols.io to enhance the reproducibility of your results. Protocols.io assigns your protocol its own identifier (DOI) so that it can be cited independently in the future. For instructions see: https://journals.plos.org/plosone/s/submission-guidelines#loc-laboratory-protocols. Additionally, PLOS ONE offers an option for publishing peer-reviewed Lab Protocol articles, which describe protocols hosted on protocols.io. Read more information on sharing protocols at https://plos.org/protocols?utm_medium=editorial-email&utm_source=authorletters&utm_campaign=protocols.

As the corresponding author, your ORCID iD is verified in the submission system and will appear in the published article. PLOS supports the use of ORCID, and we encourage all coauthors to register for an ORCID iD and use it as well. Please encourage your coauthors to verify their ORCID iD within the submission system before final acceptance, as unverified ORCID iDs will not appear in the published article. *Only* the individual author can complete the verification step; PLOS staff *cannot* verify ORCID iDs on behalf of authors.

We look forward to receiving your revised manuscript.

Kind regards,

Attila Csaba Nagy

Academic Editor

PLOS One

**Journal Requirements:**

1. If the reviewer comments include a recommendation to cite specific previously published works, please review and evaluate these publications to determine whether they are relevant and should be cited. There is no requirement to cite these works unless the editor has indicated otherwise.

2. Please review your reference list to ensure that it is complete and correct. If you have cited papers that have been retracted, please include the rationale for doing so in the manuscript text, or remove these references and replace them with relevant current references. Any changes to the reference list should be mentioned in the rebuttal letter that accompanies your revised manuscript. If you need to cite a retracted article, indicate the article’s retracted status in the References list and also include a citation and full reference for the retraction notice.

**Additional Editor Comments:**

Please move the "Statistical analysis" from "Results" to as last section of "Methods", as Reviewer 3 commented previously. The references of "Statistical analysis" used don't match the PlosOne refrence style and the rest of the manuscript. Please correct it.

[Note: HTML markup is below. Please do not edit.]

Reviewers' comments:

Reviewer's Responses to Questions

**Comments to the Author**

1. If the authors have adequately addressed your comments raised in a previous round of review and you feel that this manuscript is now acceptable for publication, you may indicate that here to bypass the “Comments to the Author” section, enter your conflict of interest statement in the “Confidential to Editor” section, and submit your "Accept" recommendation.

Reviewer #2: All comments have been addressed

Reviewer #3: (No Response)

**********

2. Is the manuscript technically sound, and do the data support the conclusions?

The manuscript must describe a technically sound piece of scientific research with data that supports the conclusions. Experiments must have been conducted rigorously, with appropriate controls, replication, and sample sizes. The conclusions must be drawn appropriately based on the data presented.

Reviewer #2: Yes

Reviewer #3: Yes

**********

3. Has the statistical analysis been performed appropriately and rigorously?

Reviewer #2: Yes

Reviewer #3: Yes

**********

4. Have the authors made all data underlying the findings in their manuscript fully available?

The PLOS Data policy requires authors to make all data underlying the findings described in their manuscript fully available without restriction, with rare exception (please refer to the Data Availability Statement in the manuscript PDF file). The data should be provided as part of the manuscript or its supporting information, or deposited to a public repository. For example, in addition to summary statistics, the data points behind means, medians and variance measures should be available. If there are restrictions on publicly sharing data—e.g. participant privacy or use of data from a third party—those must be specified.

Reviewer #2: (No Response)

Reviewer #3: Yes

**********

5. Is the manuscript presented in an intelligible fashion and written in standard English?

PLOS ONE does not copyedit accepted manuscripts, so the language in submitted articles must be clear, correct, and unambiguous. Any typographical or grammatical errors should be corrected at revision, so please note any specific errors here.

Reviewer #2: Yes

Reviewer #3: Yes

**********

6. Review Comments to the Author

Please use the space provided to explain your answers to the questions above. You may also include additional comments for the author, including concerns about dual publication, research ethics, or publication ethics. (Please upload your review as an attachment if it exceeds 20,000 characters)

**Reviewer #2:** Accept. Thank you for the modifications made to the manuscript. The introduction meets the required expectations, the analysis is sound.

**Reviewer #3:** Please move the "Statistical analysis" from "Results" to as last section of "Methods", as I commented it previously. The references of "Statistical analysis" used don't match the PlosOne refrence style and the rest of the manuscript. Please correct it.

**********

7. PLOS authors have the option to publish the peer review history of their article (what does this mean?). If published, this will include your full peer review and any attached files.

If you choose “no”, your identity will remain anonymous but your review may still be made public.

**Do you want your identity to be public for this peer review?** For information about this choice, including consent withdrawal, please see our Privacy Policy.

Reviewer #2: No

Reviewer #3: No

**********

[NOTE: If reviewer comments were submitted as an attachment file, they will be attached to this email and accessible via the submission site. Please log into your account, locate the manuscript record, and check for the action link "View Attachments". If this link does not appear, there are no attachment files.]

To ensure your figures meet our technical requirements, please review our figure guidelines: https://journals.plos.org/plosone/s/figures

You may also use PLOS’s free figure tool, NAAS, to help you prepare publication quality figures: https://journals.plos.org/plosone/s/figures#loc-tools-for-figure-preparation.

NAAS will assess whether your figures meet our technical requirements by comparing each figure against our figure specifications.

# Author comment: Author response to Decision Letter 2

**DOI:** 10.1371/journal.pone.0352255.r004

4 Jun 2026

Journal Requirements

1. If the reviewer comments include a recommendation to cite specific previously published works, please review and evaluate these publications to determine whether they are relevant and should be cited. There is no requirement to cite these works unless the editor has indicated otherwise: All the references seem justified with the manuscript.

2. Please review your reference list to ensure that it is complete and correct. If you have cited papers that have been retracted, please include the rationale for doing so in the manuscript text, or remove these references and replace them with relevant current references. Any changes to the reference list should be mentioned in the rebuttal letter that accompanies your revised manuscript. If you need to cite a retracted article, indicate the article’s retracted status in the References list and also include a citation and full reference for the retraction notice: Reviewed all the references. No changes in the current references. All the references have been checked and edited.

Editor's comments

3. Please move the "Statistical analysis" from "Results" to as last section of "Methods", as Reviewer 3 commented previously. The references of "Statistical analysis" used don't match the PlosOne refrence style and the rest of the manuscript. Please correct it.: Edited.

Attachment Submitted filename: response_to_reviewers_auresp_2.docx

# Peer review: Decision Letter 2

**DOI:** 10.1371/journal.pone.0352255.r005

**Authors:** Attila Csaba Nagy, Attila Csaba Nagy, Attila Csaba Nagy

8 Jun 2026

Tooth loss and associated factors during periodontal maintenance care in young adults with Grade C Periodontitis at a tertiary care center in Kathmandu, Nepal

PONE-D-26-00010R2

Dear Dr. Goel,

We’re pleased to inform you that your manuscript has been judged scientifically suitable for publication and will be formally accepted for publication once it meets all outstanding technical requirements.

Within one week, you’ll receive an e-mail detailing the required amendments. When these have been addressed, you’ll receive a formal acceptance letter and your manuscript will be scheduled for publication.

An invoice will be generated when your article is formally accepted. Please note, if your institution has a publishing partnership with PLOS and your article meets the relevant criteria, all or part of your publication costs will be covered. Please make sure your user information is up-to-date by logging into Editorial Manager at Editorial Manager® and clicking the ‘Update My Information' link at the top of the page. For questions related to billing, please contact billing support.

If your institution or institutions have a press office, please notify them about your upcoming paper to help maximize its impact. If they’ll be preparing press materials, please inform our press team as soon as possible -- no later than 48 hours after receiving the formal acceptance. Your manuscript will remain under strict press embargo until 2 pm Eastern Time on the date of publication. For more information, please contact onepress@plos.org.

Kind regards,

Attila Csaba Nagy

Academic Editor

PLOS One

Additional Editor Comments (optional):

Reviewers' comments:

Reviewer's Responses to Questions

**Comments to the Author**

1. If the authors have adequately addressed your comments raised in a previous round of review and you feel that this manuscript is now acceptable for publication, you may indicate that here to bypass the “Comments to the Author” section, enter your conflict of interest statement in the “Confidential to Editor” section, and submit your "Accept" recommendation.

Reviewer #3: All comments have been addressed

**********

2. Is the manuscript technically sound, and do the data support the conclusions?

The manuscript must describe a technically sound piece of scientific research with data that supports the conclusions. Experiments must have been conducted rigorously, with appropriate controls, replication, and sample sizes. The conclusions must be drawn appropriately based on the data presented.

Reviewer #3: Yes

**********

3. Has the statistical analysis been performed appropriately and rigorously?

Reviewer #3: Yes

**********

4. Have the authors made all data underlying the findings in their manuscript fully available?

The PLOS Data policy requires authors to make all data underlying the findings described in their manuscript fully available without restriction, with rare exception (please refer to the Data Availability Statement in the manuscript PDF file). The data should be provided as part of the manuscript or its supporting information, or deposited to a public repository. For example, in addition to summary statistics, the data points behind means, medians and variance measures should be available. If there are restrictions on publicly sharing data—e.g. participant privacy or use of data from a third party—those must be specified.

Reviewer #3: Yes

**********

5. Is the manuscript presented in an intelligible fashion and written in standard English?

PLOS ONE does not copyedit accepted manuscripts, so the language in submitted articles must be clear, correct, and unambiguous. Any typographical or grammatical errors should be corrected at revision, so please note any specific errors here.

Reviewer #3: Yes

**********

6. Review Comments to the Author

Please use the space provided to explain your answers to the questions above. You may also include additional comments for the author, including concerns about dual publication, research ethics, or publication ethics. (Please upload your review as an attachment if it exceeds 20,000 characters)

Reviewer #3: The authors compleres the changes I've askod. I have no further comments for the authors. Well done.

**********

7. PLOS authors have the option to publish the peer review history of their article (what does this mean?). If published, this will include your full peer review and any attached files.

If you choose “no”, your identity will remain anonymous but your review may still be made public.

**Do you want your identity to be public for this peer review?** For information about this choice, including consent withdrawal, please see our Privacy Policy.

Reviewer #3: No

**********

# Editor report: Acceptance letter

**DOI:** 10.1371/journal.pone.0352255.r006

**Authors:** Attila Csaba Nagy, Attila Csaba Nagy, Attila Csaba Nagy

PONE-D-26-00010R2

PLOS One

Dear Dr. Goel,

I'm pleased to inform you that your manuscript has been deemed suitable for publication in PLOS One. Congratulations! Your manuscript is now being handed over to our production team.

At this stage, our production department will prepare your paper for publication. This includes ensuring the following:

* All references, tables, and figures are properly cited

* All relevant supporting information is included in the manuscript submission,

* There are no issues that prevent the paper from being properly typeset

You will receive further instructions from the production team, including instructions on how to review your proof when it is ready. Please keep in mind that we are working through a large volume of accepted articles, so please give us a few days to review your paper and let you know the next and final steps.

Lastly, if your institution or institutions have a press office, please let them know about your upcoming paper now to help maximize its impact. If they'll be preparing press materials, please inform our press team within the next 48 hours. Your manuscript will remain under strict press embargo until 2 pm Eastern Time on the date of publication. For more information, please contact onepress@plos.org.

You will receive an invoice from PLOS for your publication fee after your manuscript has reached the completed accept phase. If you receive an email requesting payment before acceptance or for any other service, this may be a phishing scheme. Learn how to identify phishing emails and protect your accounts at https://explore.plos.org/phishing.

If we can help with anything else, please email us at customercare@plos.org.

Thank you for submitting your work to PLOS ONE and supporting open access.

Kind regards,

PLOS ONE Editorial Office Staff

on behalf of

Dr. Attila Csaba Nagy

Academic Editor

PLOS One
